# Predictive equations not always overestimate the resting energy expenditure in amyotrophic lateral sclerosis patients

**DOI:** 10.1186/1743-7075-8-47

**Published:** 2011-06-30

**Authors:** Guillermo P Liberé, Sabrina Guastavino, Miguel A Escobar, Eduardo L De Vito

**Affiliations:** 1 Centro del Parque, Cuidados Respiratorios, Terrada 2749, C1417CWG, Buenos Aires, Argentina

## Background

We have read with interest the paper published by Waltteri Siirala [1], in this Journal. The authors found that the measured resting energy expenditure (mREE) values were significantly lower (33.6%) in their group patient with amyotrophic lateral sclerosis (ALS) than all the predicted equations (pREE).

Significantly, the study population was in terminal stage of disease.

## Methods

Because of the small number of patients (n 5) the authors used nonparametric statistic (median and Wilcoxon). Table three shows the difference between medians (95% CI) for the gold standard mREE (kcal/d) and the equations pREE (kcal/d). The analysis of difference of medians as a statistical method for assessing agreement between two methods is misleading.

## Re-analysis

A better approach might have been plotting each value of mREE and pREE with a slope regression and identity line. As an example, according to the available data into the Siirala paper, for Harris Benedict equation the regression line would be y = 1,43x - 77,22 (r2 0,91). Slope higher than 1,0 means that higher values of kcal from the mREE are associated with greater differences than Harris Benedict values on the contrary to other papers from Sherman [2] and for our preliminary data in 10 patients with ALS [3] who also were totally dependent on invasive ventilatory support. In these reports, line regression at high values of mREE shift very closed to the identity line.

But in the analysis of measurement method comparison data, neither the correlation coefficient nor the techniques, such as regression analysis, are appropriate. Bland & Altman suggest replacing these misleading analyses by a graphical method to compare two measurement techniques. This method is simple both to do and to interpret [4]. However, a new study with much more patients will be nessesary to demostrate the advantages of this method. In fact, any statistics are not of much use (n 5) to start with. A combination of statistical tools was proposed [5].

## Conclusions

Since the indirect calorimetry is difficult to obtain, comparison between mREE and all the available equations have clinical relevance. But the differences between methods are not a constant value (bias) such as any pREE that could overestimate and underestimate kcal along the mREE.

Clearly, further studies with much more patients in several states and a proper statistic analysis are necessary in order to translate the current information to the clinical practice.

## Competing interests

The authors declare that they have no competing interests.

## References

[B1] Siirala W Olkkola KT Noponen T Vuori A Aantaa A Predictive equations overestimate the resting energy expenditure in amyotrophic lateral sclerosis patients who are dependent on invasive ventilation support Nutr Metab 2010 267 70 77 10.1186/1743-7075-7-70 PMC2939652 20796286

[B2] Sherman MS Pillai Ajay Jackson A Heiman-Patterson T Standard equations are not accurate in assessing resting energy expenditure in patients with amyotrophic lateral sclerosis JPEN 2004 28 442 446 10.1177/0148607104028006442 15568293

[B3] Liberé G Guastavino S Montemerlo H De Vito EL Cuantificación de los requerimientos calóricos en pacientes durante rehabilitación respiratoria Rev Am Med Resp 2010 Suppl 70

[B4] Bland JM Altman DG Statistical methods for assessing agreement between two methods of clinical measurement Lancet 1986 1 307 310 2868172

[B5] Weijs PJ Validity of predictive equations for resting energy expenditure in US and Dutch overweight and obese class I and II adults aged 18-65 y Am J Clin Nutr 2008 88 959 70 18842782 10.1093/ajcn/88.4.959

